# How Can Specialist Advice Influence the Neuroimaging Practice for Childhood Headache in Emergency Department?

**DOI:** 10.3390/children10121837

**Published:** 2023-11-22

**Authors:** Alberto M. Cappellari, Gaia Bruschi, Gisella B. Beretta, Maria T. Molisso, Giuseppe Bertolozzi

**Affiliations:** 1 Department of Neuroscience and Mental Health, Fondazione IRCCS Ca’ Granda Ospedale Maggiore Policlinico, 20122 Milan, Italy; maria.molisso@policlinico.mi.it; 2 Postgraduate School of Paediatrics, Università degli Studi di Milano, 20122 Milan, Italy; gaia.bruschi@unimi.it (G.B.); gisella.beretta@unimi.it (G.B.B.); 3 Pediatric Emergency Department, Fondazione IRCCS Ca’ Granda Ospedale Maggiore Policlinico, 20122 Milan, Italy; giuseppe.bertolozzi@policlinico.mi.it

**Keywords:** brain CT, brain MRI, neuroimaging, paediatrician, emergency department

## Abstract

Differentiating between primary and secondary headaches can be challenging, especially in the emergency department (ED). Since symptoms alone are inadequate criteria for distinguishing between primary and secondary headaches, many children with headaches undergo neuroimaging investigations, such as brain CT and MRI. In various studies, the frequency of neuroimaging utilization is influenced by several factors, including teaching status, ownership, metropolitan area, insurance status, and ethnicity of patients. However, only a few studies have considered the role of specialist consultations in ordering neuroimaging studies on childhood headaches. We report the contributions of different specialists to the evaluation of children with headaches admitted to the ED and their influence on neuroimaging decisions. We retrospectively reviewed the medical reports of paediatric patients who presented with headaches to the paediatric ED of the Ospedale Maggiore Policlinico of Milano between January 2017 and January 2022. Overall, 890 children with headaches were evaluated (mean age: 10.0 years; range: 1 to 17 years). All patients were examined by the ED paediatricians, while specialist consultations were required for 261 patients, including 240 neurological (92.0%), 46 ophthalmological (17.6%), and 20 otorhinolaryngological (7.7%) consultations. Overall, 173 neuroimaging examinations were required, of which 51.4 and 48.6% were ordered by paediatricians and neurologists, respectively. In particular, paediatricians required 61.4% of brain CT scans, and neurologists required 92.0% of brain MRI scans. In conclusion, paediatricians were responsible for the management of most children with headaches admitted to the ED, while specialist consultations were required only in about a third of the cases. Although there was no significant difference in the number of neuroimaging studies ordered by specialists, brain CT scans were most often used by paediatricians, and MRI scans by neurologists.

## 1. Introduction

Headache is a common symptom in children and adolescents. The prevalence of headaches ranges from 5.9 to 37.7% in children and increases to 40–50% in school-age children and 80% in adolescents [1]. Severe headaches are known to cause anxiety in both children and parents and are a common reason of visits to the paediatric emergency department (ED) [2]. Many children are admitted to the emergency department with headaches every year, accounting for approximately 1% of ED visits [3]. The primary aim of clinicians in the paediatric ED is to recognize serious life-threatening conditions requiring immediate medical care among the wide spectrum of headache diagnoses. In less severe headache types, another objective is to perform appropriate evaluations and investigations to avoid unnecessary hospitalizations [2].

Primary headaches are syndromes in which headache is not a disease process, whereas secondary headaches are disorders in which headache represents a symptom of an underlying pathological process [4]. Primary headaches in children include migraine, migraine variants, tension-type headaches, and trigeminal autonomic cephalalgias [5]. Clinical manifestations of childhood primary headaches differ from those of adults, mainly due to differences in the degree of brain maturation, including myelination, plasticity, and synaptic reorganization [5,6]. Clinical features, risk factors, and aetiologies have a strong biopsychosocial basis in childhood, distinguishing primary headache disorders in children from those in adults [6]. Therefore, diagnosing primary headaches in young children can be challenging [7]. Secondary headache disorders in children differ from those in adults [6]. Clinicians should be aware of the specific features of secondary headache disorders in children to provide effective diagnosis and management [6].

Many studies in paediatric emergency settings have reported that viral infections, particularly respiratory tract infections (pharyngitis, tonsillitis, pneumonia, sinusitis, otitis, and adenoiditis) or minor head trauma, are the most common causes of secondary headaches [2,8,9].

Although most children suffer from primary headaches or headaches caused by self-limiting diseases, a small percentage have intracranial disorders [1]. It is necessary to detect serious underlying causes, such as brain tumors, viral and bacterial meningitis, and idiopathic intracranial hypertension [2]. Many clinical features, including preschool age, recent onset of headaches, occipital location, and neurological signs, could be useful for detecting secondary headaches due to intracranial disorders [2]. To identify serious etiologies of headaches in children and avoid unnecessary testing, clinicians should obtain a thorough history, including a family history of genetic diseases predisposing to intracranial vascular malformations. Moreover, they should also evaluate the temporal pattern of headaches, inquire about the associated symptoms, investigate vital signs, examine the patient for systemic or neurological signs, and perform a fundus examination to assess for abnormalities [10]. However, distinguishing between primary and secondary headaches is difficult, particularly in an ED setting [2]. Symptoms alone are inadequate criteria for differentiating between primary and secondary headaches because patients can experience a secondary headache with the same symptoms as any primary headache [11,12,13]. The diagnosis of secondary headaches can be suggested by the presence of red flags [14], which are clinical findings that point to a possible and serious cause of headaches. However, the presence of red flags does not always indicate underlying causes that require immediate treatment, potentially misleading clinicians to waste time and resources [15]. Therefore, many children with headaches undergo neuroimaging studies such as CT and MRI [16]. Brain MRI provides better visualization and essential information but is more expensive and may require sedation, especially in children under the age of six [17]. On the other hand, CT scans expose paediatric patients to radiation, increasing the risk of malignancy later in life [18,19]. Because of cranial bone marrow production, age-dependent risk is especially high in younger children [19,20].

When deciding whether to perform an imaging study in children with headaches, risk stratification based on clinical history and physical examination should be considered to ensure that the benefits of neuroimaging exceed the risks of radiation exposure from CT scans or sedation for MRI [21]. However, defining reliable red flags for the prediction of severe disorders and, consequently, validating criteria for the appropriate use of neuroimaging during diagnostic workup are challenging [22]. Many factors, such as teaching status, ownership, metropolitan area, insurance status, and ethnicity of patients, affect how frequently CT is used in various studies [23]. Among the studies reporting specialist consultations in patients with headaches admitted to the ED [24,25], only a few authors have considered the role of different specialists in ordering neuroimaging investigations in children with headaches [26].

The aim of our paper was to evaluate how specialist advice can influence the choice of neuroimaging studies in the paediatric ED.

## 2. Materials and Methods

This retrospective study included children with headaches admitted to the paediatric ED of Ospedale Maggiore Policlinico of Milano between January 2017 and January 2022. The same population was the subject of a recently published study approved by the ethics committee of our hospital [27]. The Inclusion criteria were age < 18 years, admission to the paediatric ED for headaches, and a lack of verbal or developmental delays. Patients who were unable to provide details about their headaches, such as the location or sudden onset, were excluded from this study. Very young children were also excluded unless their parents could provide such information. For patients who had more than one access to the ED due to headaches during the study period, only the most recent admission was considered.

The following data were evaluated for each patient: demographic characteristics, personal history of headache, clinical characteristics, underlying disorders, and the results of diagnostic procedures, including neuroimaging and laboratory testing. Headaches were classified into three categories: primary headaches (headaches without underlying medical conditions), secondary headaches (headaches associated with underlying medical conditions), and headaches of unknown aetiology. Secondary headaches were further grouped into intracranial (meningoencephalitis, cerebrovascular disorders, and structural abnormalities) and non-intracranial disorders (systemic infections, toxic-metabolic disorders, ocular diseases, otorhinolaryngological diseases, and functional disorders).

Medical consultations included visits to paediatricians, neurologists, and other specialists, such as otolaryngologists and ophthalmologists. The total number of consultations was recorded, as well as the various specialists involved. Furthermore, the number of neuroimaging investigations required by different specialists was assessed.

Excel was used for data collection, and statistical analysis was performed with R 4.3.0 (R Core Team Software, Vienna, Austria). Continuous variables were reported as mean and range values, while categorical variables were presented as frequencies and percentages. The Chi-square test or Fisher’s exact test for small counts was used to compare the medical consulting and neuroimaging findings. Statistical significance was set at 5%.

## 3. Results

During the 5 years period of the study, 890 children with headaches were evaluated in our paediatric ED (mean age: 10.0 years; range: 1 to 17 years). The demographic data of the study population are summarized in Table 1. Most patients were discharged (90.8%, 808 patients), whereas the remaining patients were hospitalized (9.2%, 82 patients).

All patients underwent paediatric visits by the ED paediatricians. Overall, 261 specialist consultations were requested in the ED, including 240 neurological (92.0%), 46 ophthalmological (18.0%), and 20 otorhinolaryngologists (7.7%) consultations. Two specialist consultations were requested for 46 patients (17.6%), whereas only 1 patient (0.4%) received all three consultations (Table 2).

Headaches were located frontotemporally in 611 patients (68.7%), occipital in 52 (5.8%), diffuse in 107 (12.0%), and unspecified in 120 (13.5%).

Primary headaches occurred in 337 (37.9%) patients, secondary headaches in 353 (39.7%) patients, and headaches of unknown aetiology in 200 (22.5%). Primary headache was diagnosed by paediatricians and neurologists in 43.5% and 56.5% of the patients, respectively. Paediatricians diagnosed secondary headaches in 65.6% of cases (intracranial diseases in 16 patients and non-intracranial disorders in 26 patients), whereas neurologists diagnosed secondary headaches in 34.4% of cases (intracranial diseases in 9 patients and non-intracranial disorders in 13 patients). All patients with headaches of unknown aetiology were visited by paediatricians and neurologists.

Overall, 173 neuroimaging examinations were performed, with 89 scans ordered by a paediatrician (51.4%) and 84 by a neurologist (48.6%). In particular, paediatricians required 81 out of 132 brain CT scans (61.4%), while neurologists ordered 23 out of 25 brain MRIs (92.0%). Both CT and MRI were performed in 16 patients, and most scans (62.5%) were ordered by neurologists (Table 3). Abnormalities were found in 27 of the 173 neuroimaging examinations performed (15.6%).

## 4. Discussion

Neurological disorders represent a relevant component of paediatric emergencies, involving up to 20 to 40% of children with the highest severity codes at triage evaluation in the paediatric ED [28]. Epileptic and non–epileptic events and headaches account for over two–thirds of the cases [22]. In certain centers, over the last decade, the rate of paediatric ED visits for non–traumatic headaches has increased from 63.6 to 166% [29,30]. The high frequency of emergency clinical presentations is associated with an increasing need for specialized neuropaediatric consultants [22]. Consultations with specialists in ED are required for safe and effective patient care [31]. This process should result in a variety of outcomes, including the completion of procedures or investigations, as well as hospitalization or discharge [32,33,34,35]. Some studies have reported specialist consultations in patients with headaches admitted to the ED [24,25]. Still, only a few authors have investigated the role of different specialists in ordering neuroimaging investigations for children with headaches [26].

Our study showed that emergency paediatricians were the sole clinicians in charge of most children with headaches admitted to the ED, with specialist consultations required in only approximately one–third of the cases. This finding is consistent with those reported in other Italian studies, in which the rate of specialist visits ranged from 28.2% to 49.7% [24,25]. Therefore, we cannot exclude the possibility that the hospital’s organization, patient triage method, and intra–hospital protocols used in our country played a role in explaining these results. According to the literature, most secondary headaches in our patients were benign and self-limiting [2,3,24,26,36,37,38], and they were usually managed by ED paediatricians. Nonetheless, paediatricians were also frequently involved in the diagnosis of headaches associated with intracranial disorders, whereas neurologists were most often responsible for the diagnosis of primary headaches. This finding could be partially explained by the difficulty of establishing a definitive diagnosis in children during their first visit because the diagnosis of primary headache is easily made when certain International Headache Society (IHS) criteria are fulfilled [39].

The first step in the evaluation of a child or adolescent with a headache is to distinguish between primary and secondary headaches [21]. Although most childhood headaches are benign, parents are frequently concerned about the possibility of a brain tumour or vascular malformation, especially if there is a family history of congenital aneurysms [17]. Clinicians should obtain detailed clinical history and perform comprehensive clinical examinations. The presence of provoking factors associated with migraine or tension-type headache, such as stress or sleep deprivation, as well as recent head trauma, fever, or features associated with systemic disease, suggesting secondary headaches, should be considered in the clinical history [5]. It is essential to evaluate the temporal pattern of headaches because acute or chronic progressive headaches are usually associated with secondary disorders. In contrast, episodic or chronic nonprogressive headaches may suggest a primary headache [40].

Family history should be investigated for the presence of primary or secondary headaches in other family members, including brain tumours, vascular disorders, and autoimmune diseases [5]. In all patients with headaches, detailed general and neurological examinations should be performed, including a search for meningismus, assessment of the cranial nerves, and examination of the optic discs [5,41,42]. Symptoms alone cannot distinguish between primary and secondary headaches since patients with secondary headaches may experience the same symptoms as those with primary headaches [11,12,13]. Nonetheless, the main goal of the clinical history and examination is to look for “red flags” [21], which are clinical findings that point to a possible and serious cause of headaches, compelling physicians to perform advanced investigations [15]. Certain red flags are more common in patients with secondary headaches [43], and patients with one or more red flags are at a higher risk of underlying intracranial diseases [39]. The main red flags included the following findings: (1) abnormal neurologic examination; (2) associated symptoms such as vertigo, intractable vomiting, mental status changes, focal neurologic signs, or systemic symptoms; (3) headache waking the child from sleep; (4) occipital headache; (5) first or worst headache; (6) recent headache of less than 6 months duration; (7) change in type of headache; (8) subacute onset and progressive headache; (9) new-onset headache in a child with immunosuppression; and (10) no family history of migraine or primary headaches. Although red flags act as screening tools that help physicians identify those patients with headaches who would benefit from immediate neuroimaging [44], they do not always indicate that the underlying cause requires emergency treatment [15]. Among the cases requiring specialist consultation, we found that neurologists were involved in over 90% of the cases, which is consistent with the results of other studies on headaches, in which the neurologist was the most frequently consulted specialist [25,26]. In a study of adults admitted to the ED for headaches by Relja et al., neurological visits accounted for 81.2% of specialist consultations and 12.7% of all neurological consultation visits to the ED during the same period [25]. In a study of 1833 patients admitted to the paediatric ED with headache, Rossi et al. requested 390 specialist consultation visits, including 187 neurological, 156 ophthalmological, 19 neurosurgical, and 28 otorhinolaryngologic consultations [26]. Headache is one of the main reasons for requiring an urgent neurological visit to the paediatric ED [25], and patients admitted to the ED for headaches have a high chance of being diagnosed with a secondary disorder [26]. The rarity of ophthalmologic and otorhinolaryngological consultations in our ED seems to be consistent with those of other studies [24,26]. Since headaches caused by ophthalmological problems are more frequently chronic, they are less common in emergency settings [24]. Although otorhinolaryngological consultation may be necessary if recurrent and chronic sinusitis is suspected [44], a diagnosis of acute uncomplicated sinusitis should be based solely on the clinical history and physical examination without the need for imaging [21].

Previous studies have reported that 6.3% to 44% of children admitted to the ED with headaches undergo neuroimaging studies [3,24,45,46,47,48,49]. The wide range of imaging rates reflects a lack of agreement or formal recommendations on the management of children and adolescents with headaches in the ED [26]. The decision to order imaging studies for children with headaches should involve risk stratification based on clinical history and physical examination to assess the benefits and risks of neuroimaging [21].

Brain CT is highly sensitive for detecting intracranial hemorrhage but less sensitive for evaluating intracranial masses and infections. Therefore, CT is appropriate for sudden severe headaches because their aetiology might be related to a ruptured aneurysm or arteriovenous malformation [50,51,52]. However, because brain CT exposes paediatric patients to radiation, increasing the risk of malignancy later in life [18,19], its use is restricted to specific cases [21]. Although brain MRI is the preferred neuroimaging technique for children and adolescents due to the lack of radiation exposure, it is not without risks, such as sedation or general anaesthesia [21]. Since headache is the only symptom of 1% of paediatric brain abnormalities [53,54,55,56], neuroimaging is considered unnecessary in patients with headaches who have no other neurologic symptoms, suggesting a serious intracranial disorder [54,57]. Nonetheless, overuse of CT [58] and MRI in paediatric ED has been reported, and there is a need for current studies covering paediatric imaging overuse trends, usage variability, and adherence to clinical protocols [59]. Although the American Academy of Neurology (AAN) Practice Guidelines recommended a diagnostic approach for children and adolescents with recurrent headaches, they made no specific statement about patients admitted to the ED for headaches [50].

Our study showed no significant difference in the number of neuroimaging studies required by paediatricians and neurologists. In contrast, Rossi et al. reported that emergency paediatricians ordered 63.5% of the neuroimaging investigations required for headaches in paediatrics, a neurologist ordered 30.4%, and another consultant ordered 4.8% [26]. However, we found that brain CT was most often ordered by the paediatricians and MRI by the neurologists. The outcomes observed in our study can be explained by several factors. First, in an emergency situation, paediatrician must quickly rule out life-threatening conditions that are frequently detected on brain CT scans. CT can be useful in this setting because it does not require sedation and is faster for acquiring brain images, although radiation exposure from CT scans in childhood has been linked to an increased risk of leukemia and brain tumors [19].

Second, even though brain MRI use in the emergency setting has recently increased due to the desire to provide radiation-free imaging, performing an MRI in the paediatric ED is still uncommon due to the need for sedation in very young children [60]. It is interesting to note that in paediatric patients with recurrent headache, provider specialty was associated with the likelihood of receiving a CT scan, with a neurologist being associated with a lower likelihood of a child undergoing a CT scan than a family physician [60].

Despite the fact that many patients have been seen by a paediatric neurologist, the frequency of headaches of unknown origin is high in our study. Indeed, we defined our cohort based on the primary discharge diagnosis without a specific follow-up program for these patients, which could have been useful for reclassifying headaches of unknown aetiology.

## 5. Conclusions

The diagnostic dilemma in evaluating children and adolescents with headaches is that most do not have intracranial disorders. Still, a small percentage of serious disease disorders may manifest clinically as isolated headaches. Therefore, many paediatric patients undergo brain CT and MRI scans [16]. Since CT uses ionizing radiation at doses that predispose patients, especially children, to malignancies [19], clinicians should weigh the risk of causing harm against the possibility of missing a serious disorder [61]. Reducing radiation exposure during the treatment of common diseases is a priority in the ED [62,63]. There are ongoing efforts to reduce radiation exposure through age and weight adjustments, as well as novel CT technologies [64].

Nonetheless, ED clinicians should use patient history or physical examination findings to establish the risk of an emergent intracranial abnormality in a child with a headache and to decide whether to order emergent brain imaging [50,51,65,66,67,68,69]. Several clinical evidence-based guidelines for children with headaches can help identify the patients who are more likely to have serious underlying causes for their headaches [16]. Although practice guidelines can be effective, they are not universally adopted, and the risk of missing an intracranial lesion persists even when followed [61]. Brain MRI use and availability in the ED have increased in recent years due to the desire to use radiation-free imaging [70] and reduce the length of stay in the ED until MRI imaging becomes available [60]. MRI techniques are evolving to detect intracranial disorders in children without exposing them to radiation risks, and rapid-sequence brain MRI protocols have been developed for paediatric patients [62,71].

The novelty of our study lies in defining the role of specialist consultations in the management of childhood headaches in the ED. Children with headaches admitted to the ED are first examined by a paediatrician, who is ultimately responsible for deciding whether to perform neuroimaging studies. The emergency paediatrician prefers brain CT to rule out life-threatening intracranial disorders in emergency settings. Neurologic consultation may be more useful in the diagnosis of primary or secondary headaches associated with a normal CT scan. In recent years, there has been a trend toward more conservative use of CT imaging in an attempt to limit paediatric patients’ exposure to ionizing radiation [63]. A closer collaboration between emergency paediatricians and other ED specialists could improve this outcome.

## Figures and Tables

**Table 1:** Demographic data of 890 patients with headaches admitted to the ED.

Demographic Data		
Age (years)—mean (range)		10 (1–17)
Sex—N (%)	Male	430 (48.3)
	Female	460 (51.7)
Pre-existing headache—N (%)	Yes	316 (35.9)
(881/890, 99.0%)	Primary headache	224 (70.9)
	Secondary headache	44 (13.9)
	Unknown headache	48 (15.2)
	No	565 (64.1)
Underlying conditions—N (%)	No	795 (89.3)
	Systemic diseases	50 (5.6)
	Genetic neurological diseases	17 (1.9)
	Acquired neurological diseases	28 (3.1)

**Table 2:** Number of medical consultations.

Medical Consultations	Total Number (%)
Paediatrician	890 (100.0)
Paediatrician only	629 (70.7)
Specialist	261 (29.3)
Neurological	240 (92.0)
Otorhinolaryngological	20 (7.7)
Ophthalmological	46 (17.6)

**Table 3:** Number of neuroimaging investigations according to medical consultations.

	Patients with Imaging N (%) (n = 173)	Brain CT N (%) (n = 132)	Brain MRI N (%) (n = 25)	Brain CT + MRI N (%) (n = 16)	*p*
Paediatrician	89 (51.4)	81 (61.4)	2 (8.0)	6 (37.5)	<0.001
Neurologist	84 (48.6)	51 (38.6)	23 (92.0)	10 (62.5)	

Legend. The *p*-value refers to the result of the Fisher exact test applied to the 2 × 3 contingency table obtained by comparing the variables “medical consultations” (paediatrician, neurologist) and “neuroimaging” (brain CT, brain MRI, brain CT + MRI).

## Data Availability

The data presented in this study are available upon request from the corresponding author.
